# Oxygenation of the Intraportally Transplanted Pancreatic Islet

**DOI:** 10.1155/2016/7625947

**Published:** 2016-10-30

**Authors:** Thomas M. Suszynski, Efstathios S. Avgoustiniatos, Klearchos K. Papas

**Affiliations:** ^1^Department of Surgery, University of Minnesota, Minneapolis, MN 55455, USA; ^2^Institute for Cellular Transplantation, Department of Surgery, University of Arizona, Tucson, AZ 85724, USA

## Abstract

Intraportal islet transplantation (IT) is not widely utilized as a treatment for type 1 diabetes. Oxygenation of the intraportally transplanted islet has not been studied extensively. We present a diffusion-reaction model that predicts the presence of an* anoxic* core and a larger* partly functional* core within intraportally transplanted islets. Four variables were studied: islet diameter, islet fractional viability, external oxygen partial pressure (*P*) (in surrounding portal blood), and presence or absence of a thrombus on the islet surface. Results indicate that an islet with average size and fractional viability exhibits an anoxic volume fraction (AVF) of 14% and a function loss of 72% at a low external *P*. Thrombus formation increased AVF to 30% and function loss to 92%, suggesting that the effect of thrombosis may be substantial. External *P* and islet diameter accounted for the greatest overall impact on AVF and loss of function. At our institutions, large human alloislets (>200 *μ*m diameter) account for ~20% of total islet number but ~70% of total islet volume; since most of the total transplanted islet volume is accounted for by large islets, most of the intraportal islet cells are likely to be anoxic and not fully functional.

## 1. Introduction

Islet transplantation (IT) remains a promising therapy for diabetes mellitus but current results justify its clinical use only with a small subset of type 1 diabetics. In 2000, the Edmonton protocol (EP), which recommended the intraportal transplantation of >10,000 islet equivalents (IE) per kilogram recipient body weight (kgBW) with a specialized steroid-free immunosuppressive protocol (low-dose tacrolimus, sirolimus, and IL-2 receptor antibody induction), enabled consistent diabetes reversal and short-term (<1-year) insulin independence [1–3]. These results were replicated at other institutions [4, 5], but long-term (>5 year) outcomes on the EP were poor [6]. Despite being a major breakthrough, the EP often required donor islets isolated from 2–4 pancreata. This requirement of multiple pancreas donors is a major limitation that prohibits widespread availability of IT due to increased costs and clinical risk associated with multiple procedures, placing an additional strain on an already limited donor pancreas supply. In the mid-2000s, new trials were undertaken to establish protocols that enable successful IT using islets from a single donor pancreas [7, 8]. Newer induction immunosuppressive agent combinations [T-cell-depleting antibody (anti-CD3 antibody, alemtuzumab, or antithymocyte globulin) and a tumor necrosis factor alpha (TNF-*α*) inhibitor (etanercept or infliximab)] have improved long-term diabetes reversal rates (~50% in 5 years at the most experienced centers) [9], presumably by preserving transplanted *β*-cell mass. However, allo-IT cannot yet be offered to all type 1 diabetics. It remains unclear whether immunologic or nonimmunologic causes are primarily responsible for the gradual attrition of insulin independence with time.

Many investigators believe that the liver may not be the optimal IT site and there are numerous reasons [10], including, but not limited to, (a) intraportal thrombus formation on the islet surface, complement-mediated islet cell lysis, and local inflammation [11], sometimes collectively referred to as the “instant blood-mediated inflammatory reaction,” which are believed to contribute to alloimmune rejection and early islet loss [12, 13]; (b) possible immediate exposure to islet cell-directed T memory cells and recurrence of autoimmune rejection [14]; (c) higher local concentrations of orally administered immunosuppressants [15], which can impair insulin secretion or islet revascularization [16, 17]; (d) slow reestablishment of surrounding extracellular matrix, which can adversely affect islet survival [18, 19]; (e) inability to easily track, image, or retrieve the graft [20, 21]; and (f) poor oxygenation due to the mixed portal circulation, significant oxygen gradients within the hepatic tissue [22], and slow and possibly incomplete revascularization [23–26], which is of particular importance since insulin-secreting *β*-cells are not designed to function under conditions of hypoxia [27].

Poor oxygenation, particularly in the early post-IT period and before revascularization occurs, is not highly appreciated and has not been well studied by the field. An assumption made is that local oxygen partial pressure (*P*) must be adequate for islet survival and function simply because islets are in direct contact with the blood stream. However, there are major differences in the route and mechanism of oxygen delivery when comparing a native islet in a healthy pancreas and an intraportally transplanted islet. First, native islets are highly perfused in part with oxygen-saturated arterial blood, receiving 15–20% of total blood flow to the pancreas [28] despite accounting for 1-2% of the total pancreatic volume [29], but intraportal islets have no blood perfusion starting at the time of organ procurement and from that point on rely on oxygen diffusion from their surface until they are revascularized. Second, most native islet cells are within the distance of a single cell (~10–15 *μ*m) from the nearest oxygen source [30], whereas cells in a transplanted islet can be >200 *μ*m away from the nearest oxygen source or further if there is thrombus formation. Moreover, as islets lodge in the distal hepatic sinusoids, the distal half of the islet is not in direct contact with the bloodstream. These diffusion distances reduce the availability of oxygen to the intraportal islet cells, especially those located within the core of the islet, which may affect islet cell survival and function. For example, it was found that stimulated insulin secretion rate can be halved at *P* in the bulk perfusate below 40 mm Hg [31]. The range of *P* in the intraportal blood (*P*_ext_) is estimated to be 5–40 mm Hg [32, 33]. In this setting, low *P*_ext_, in combination with increased oxygen diffusion distances and with the possible formation of a surface thrombus which imposes additional oxygen transfer resistance, can significantly reduce the viability and function in an intraportally transplanted islet.

To quantify the effects of limited oxygenation, we developed a model that predicts the presence of an anoxic core, a larger, partially functional core within intraportally transplanted islets, and the concomitant functional loss of insulin secretory capacity. We present our findings and also discuss the potential implications for clinical IT.

## 2. Methods

**Figure 1:**
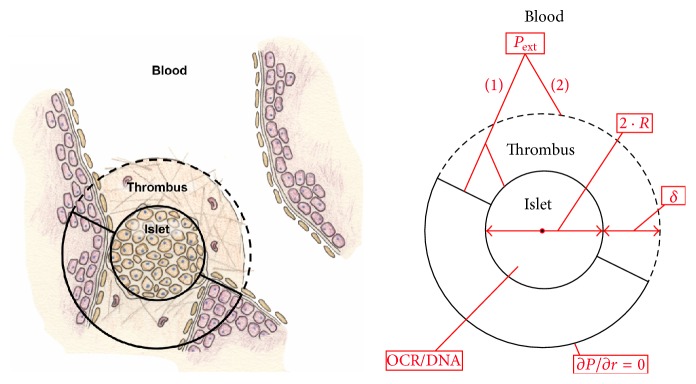
Schematic depicting the intraportal islet which is modeled as a spherical body containing viable oxygen-consuming cells. The transplanted islet is lodged at a bifurcation in a distal hepatic sinusoid and has access to portal blood at its proximal half-surface. The distal half of the islet equilibrates with the surrounding environment and is modeled by the presence of a no-flux boundary condition at a specified distance away from its back surface (∂*P*/∂*r* = 0). There are 4 parameters that are adjusted in this model, including (1) fractional viability, or oxygen consumption rate normalized to DNA content (OCR/DNA); (2) islet diameter (2 · *R*); (3) external blood *P* (*P*_ext_); and (4) presence or absence of thrombus with a specified thickness (*δ*), located only on the proximal half-surface.

Figure 1 depicts a schematic that illustrates the generalized model geometry, key variables, and boundary conditions. An islet was assumed to be a spherical body of homogeneous tissue that is lodged near a bifurcation of a distal hepatic sinusoid. *P*_ext_ was assumed to be constant and with no gradients across the interface between the blood and the surrounding tissue in the proximity of a single islet. The blood was assumed to bathe the proximal half of the islet itself or a hemispherical non-oxygen-consuming thrombus surrounding the proximal half of the islet. The distal half of the islet was assumed to be in contact with non-oxygen-consuming homogeneous host tissue with a zero oxygen flux boundary condition (∂*P*/∂*r* = 0) applied at a distance of 100 *μ*m from the islet surface. When initially evaluating the model, the no-flux boundary condition was adjusted to note that changes in its distance did not affect the model results significantly. The time scale for changes in the islet geometry (e.g., due to loss of viability in the anoxic core) is much larger than the characteristic time for diffusion, thus establishing a pseudo-steady-state. Under these assumptions, oxygen transport is by diffusion alone and can be described by the steady-state oxygen diffusion-reaction equation:

(1) $$\begin{array}{c} {\left(\;\alpha D\;\right)}_{i}\nabla^{2}P=V_{O_{2}}, \end{array}$$

where (*αD*)_*i*_ is the oxygen permeability (which is the product of oxygen solubility and diffusion coefficients) within each domain *i* (islet, thrombus, or tissue surrounding the islet), ∇^2^ is the Laplacian operator (which represents the second-derivative with respect to all three spatial dimensions), and *V*_O_2__ is the rate of oxygen consumption per unit volume. Since the oxygen partial pressure in the blood is assumed to be uniform, no azimuthal variation is evident from the geometry. The model is two-dimensional and axisymmetric and was solved as such using the finite element COMSOL Multiphysics software (Burlington, MA).

We obtained most model parameters from literature. All values or ranges for model parameters used in this study are presented in Table 1 along with references. There are four parameters in this model that were studied within ranges that are reasonable based on prior experimental evidence:

(a) Islet fractional viability, expressed in terms of oxygen consumption rate (OCR) per DNA content (OCR/DNA) of islet tissue.
(b) Islet diameter (2 · *R*).
(c) External *P* in the blood near the islet (*P*_ext_).
(d) Presence or absence of thrombus (with specified thickness, *δ*).

The OCR of the islet was modeled using Michaelis-Menten kinetics:

(2) $$\begin{array}{c} V_{O_{2}}={\left(\;V_{O_{2}}\;\right)}_{max}\cdot \frac{P}{K_{m}+P}, \end{array}$$

where (*V*_O_2__)_max_ [mol/cm^3^/s] is the maximum volumetric islet OCR and *K*_m_ [mm Hg] is the Michaelis-Menten constant for islet oxygen consumption, which is estimated to be 0.44 mm Hg [39]. We used a representative range of values for OCR/DNA [nmol/min/mg DNA] obtained via characterization of *β*-cells [42], porcine islets [43], and human auto- and alloislets using the stirred microchamber system under non-oxygen-limited conditions [44, 49]. The unit conversion between OCR/DNA and (*V*_O_2__)_max_ was done by assuming that 1 IE contains 10.4 ng of DNA [50].

To model an anoxic core within the islet, a critical local *P* value (*P*_*C*_ = 0.1 mm Hg) at which islet tissue ceases to consume oxygen and becomes nonviable was used based on experimental evidence [37, 39]. Using this model, the anoxic volume fraction (AVF) is calculated using

(3) $$\begin{array}{c} AVF=\frac{{\left.V\right|}_{P=P_{C}}}{\left(4\pi /3\right)R^{3}}, \end{array}$$

where the numerator is the numerically calculated anoxic islet volume and *R* is the islet radius.

To model loss of insulin secretory capacity, we used an* ad hoc* model [39] for the local second-phase glucose-stimulated insulin secretion rate per unit islet volume, *S*, given by

(4) S=0,P=PCS=SmaxPP∗,PC<P<P∗S=Smax,P∗≤P,

where *S*_max_ is the value of *S* under non-oxygen-limiting conditions and *P* ^*∗*^ is another critical value of local *P* below which *S* becomes limited by hypoxia. The value of *P* ^*∗*^ was derived by fitting second-phase glucose-stimulated insulin secretion data by rat islets available at different bulk perfusate *P* [31, 34, 40] to a diffusion-reaction model similar to the one used in this study that also took into consideration the *P* gradient through the boundary layer around the islets and that *β*-cells are not homogeneously distributed throughout the rat islet [34, 39]. Using the model described by (4), the fractional loss of insulin secretory capacity (FLISC) can be calculated by

(5) $$\begin{array}{c} FLISC=1-\frac{\int_{V}{\left.\min\left(\left(P/P^{*}\right),1\right)\right|}_{P>P_{C}}dV}{\left(4\pi /3\right)R^{3}}. \end{array}$$

To illustrate the potential impact of early islet oxygenation on an entire alloislet preparation, actual islet size distribution data estimated via standard light microscopy from 23 clinical human alloislet preparations (high-purity, cultured prior to transplant) prepared at the University of Minnesota (5/19/2009–1/8/2012) were used. Islets were segregated into estimated size ranges (e.g., with diameters of 50–100 *μ*m, 100–150 *μ*m, etc.) following measurements of representative aliquots using standard light microscopy. The percentage of islets found within each measured size range was referred to as a “number fraction.” Number fractions were then converted to volume fractions by having each islet estimated to have a diameter which was the mean of each size range (e.g., 75 *μ*m diameter for an islet allocated to the size range with diameter somewhere within 50–100 *μ*m), calculating the total volume of all islets within each size range by assuming that each islet is spherical, and then dividing the total islet volume within each size range by the total islet volume in the entire preparation.

## 3. Results

**Figure 2:**
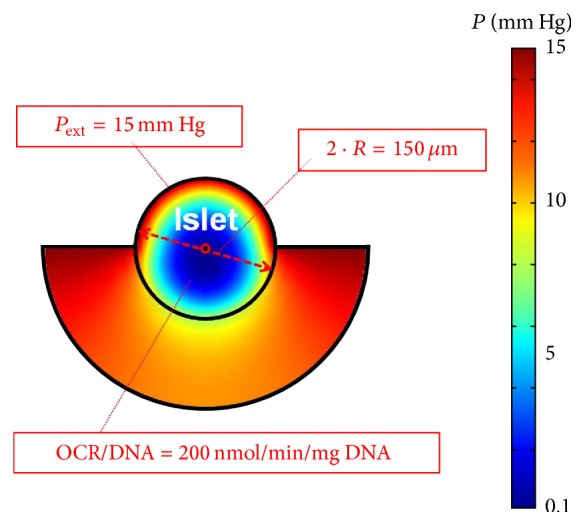
Surface plot illustrating model results for a “*Baseline Case,*” which involves an islet with average diameter (2 · *R* = 150 *μ*m) and fractional viability (OCR/DNA = 200 nmol/min/mg DNA), exposed to a reasonable oxygen supply (*P*_ext_ = 15 mm Hg), and no thrombus formation at its proximal half-surface (*δ* = 0 *μ*m). The colors within and behind the islet depict the calculated spatial *P* gradients, as indicated by the legend (right). *δ*, thickness of the thrombus; OCR, oxygen consumption rate; *P*, oxygen partial pressure; *P*_ext_, external blood *P*; *R*, islet radius.

**Figure 3:**
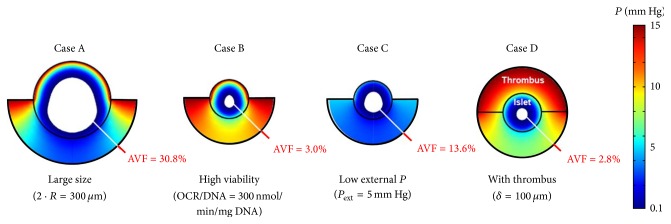
Surface plots illustrating model results for four different cases in which the* Baseline Case* (no thrombus, OCR/DNA = 200 nmol/min/mg DNA, *P*_ext_ = 15 mm Hg, and 2 · *R* = 150-*μ*m) is perturbed by adjusting only 1 of the 4 parameters with each case.* Case A* depicts the* Baseline Case* with an increase in the islet diameter from 150 to 300 *μ*m.* Case B* depicts the* Baseline Case* with an increase in the OCR/DNA from 200 to 300 nmol/min/mg DNA.* Case C* depicts the* Baseline Case* with a decrease in *P*_ext_ from 15 to 5 mm Hg.* Case D* depicts the* Baseline Case* with the addition of a 100 *μ*m thrombus on the proximal half-surface of the islet. The anoxic volume fraction (AVF) is depicted by the achromatic core. Note the magnitude of the AVF associated with each perturbation. AVF, anoxic volume fraction; OCR, oxygen consumption rate; *P*, oxygen partial pressure; *P*_ext_, external blood *P*; *R*, islet radius.

**Figure 4:**
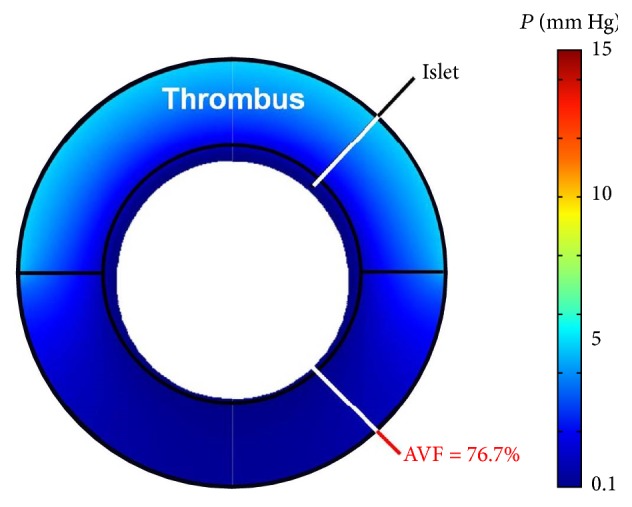
Surface plot illustrating model results for the worst case scenario (*Worst Case*) analyzed in this study, which combines the* Baseline Case* and the 4 individual perturbations of 4 parameters (increase in islet diameter, increase in fractional viability, decrease in *P*_ext_, and addition of thrombus) that were shown separately in Figure 3. Note the very large anoxic volume fraction (AVF). AVF, anoxic volume fraction; *P*, oxygen partial pressure.

We calculated the AVF and the FLISC for intraportally transplanted islets with different properties (size, fractional viability) and in different local environments (*P*_ext_, ± thrombosis). For *P* ^*∗*^ we used the best case scenario value of 5 mm Hg. For illustration, we consider a* Baseline Case*: an intraportal islet of average size (2 · *R* = 150 *μ*m) and fractional viability (OCR/DNA = 200 nmol/min/mg DNA), exposed to a reasonable but low *P*_ext_ (15 mm Hg), and with no thrombus formation at its proximal half-surface (Figure 2). In this case, the calculated AVF is 0 and the FLISC is 13%. The relative impact of thrombus formation, *R*, OCR/DNA, and *P*_ext_ can be examined by adjusting model parameters individually (Figure 3). If the same intraportal islet described by the* Baseline Case* is larger in size (2 · *R* = 300 *μ*m), then a very large anoxic core (AVF = 30.8%) and functionality loss (FLISC = 63%) are predicted (*Case A*). If the islet from the* Baseline Case* is highly viable and has a greater OCR/DNA (300 nmol/min/mg DNA), then a small anoxic core (AVF = 3.0%) and a significant functionality loss (FLISC = 29%) are predicted (*Case B*). If the same islet from the* Baseline Case* is exposed to a lower *P*_ext_ (5 mm Hg), then a larger anoxic core (AVF = 13.6%) and a very large functionality loss (FLISC = 72%) are predicted (*Case C*). Finally, if the same islet from the* Baseline Case *has a 100 *μ*m thick thrombus form on its proximal half-surface, then a small anoxic core (AVF = 2.8%) and a much larger functionality loss (FLISC = 42%) are predicted (*Case D*). If all the single parameter perturbations (*Cases A–D*) are combined so that the islet proposed in the* Baseline Case* now has a thrombus on its proximal half-surface, is of higher fractional viability (OCR/DNA = 300 nmol/min/mg DNA) and size (2 · *R* = 300 *μ*m), and is exposed to a very low *P*_ext_ (5 mm Hg), then most of the islet is predicted to be anoxic (AVF = 76.7%) and the islet is effectively not secreting insulin (FLISC = 98%); this represents the worst case scenario examined from the standpoint of oxygenation (Figure 4,* Worst Case*).

**Figure 5:**
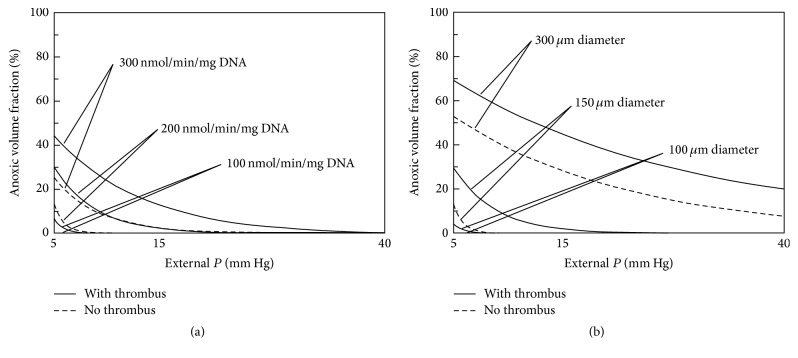
Graphs depicting a summary of model results for calculation of anoxic volume fraction [AVF (%)] with respect to external blood *P* (*P*_ext_), islet fractional viability (OCR/DNA), and diameter (2 · *R*) and with or without thrombus formation (*δ* = 100 *μ*m). The graph on the left (a) illustrates the change in AVF for an islet of average diameter (150 *μ*m) for the 3 OCR/DNA values. The graph on the right (b) illustrates the change in AVF in an islet with average OCR/DNA (200 nmol/min/mg DNA) for 3 islet diameter values. AVF is defined as the region of the islet that is anoxic, occurring below a critical *P* (*P*_*C*_) of 0.1 mm Hg. AVF, anoxic volume fraction; *δ*, thickness of the thrombus; OCR, oxygen consumption rate; *P*, oxygen partial pressure; *P*_*C*_, critical *P* for viability; *P*_ext_, external blood *P*; *R*, islet radius.

**Figure 6:**
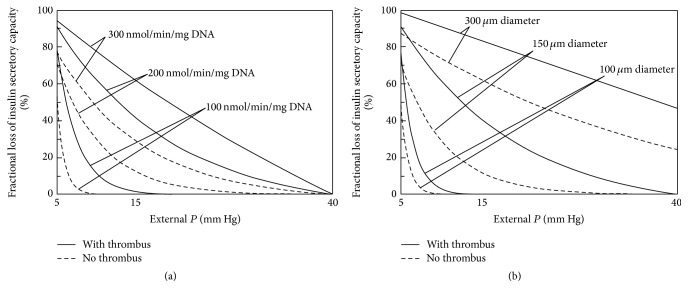
Graphs depicting a summary of model results for calculation of fractional loss of insulin secretory capacity [FLISC (%)] with respect to external *P* (*P*_ext_), islet fractional viability (OCR/DNA), and diameter (2 · *R*) and with or without thrombus formation (*δ* = 100 *μ*m). The critical *P* ^*∗*^ used in the model was 5 mm Hg, which represents the best case scenario. The graph on the left (a) illustrates the change in FLISC for an islet of average diameter (150 *μ*m) for the 3 OCR/DNA values. The graph on the right (b) illustrates the change in FLISC for an islet with average OCR/DNA (200 nmol/min/mg DNA) for 3 islet diameter values. FLISC is defined as the loss of insulin secretory capacity relative to an islet not limited by hypoxia. FLISC, fractional loss of insulin secretory capacity; *δ*, thickness of the thrombus; OCR, oxygen consumption rate; *P*, oxygen partial pressure; *P* ^*∗*^, critical *P* for insulin secretion; *P*_ext_, external blood *P*; *R*, islet radius.

The relative impact of islet fractional viability and size is further examined by plotting the AVF (Figure 5) and FLISC (Figure 6) with parameter *P*_ext_ (5–40 mm Hg), with or without thrombus formation. Within the range of islet OCR/DNA values and diameters examined, it appears that islet size more strongly affects the magnitude of both AVF and FLISC. Furthermore, the formation of a thrombus may markedly affect islet cell survival and function.

**Figure 7:**
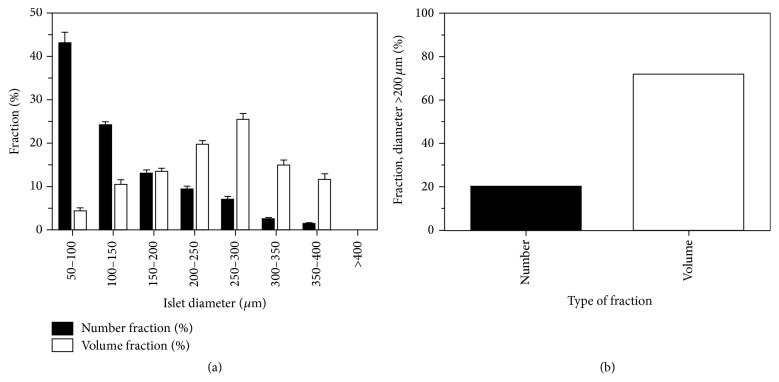
Islet size distribution stratified by ranges of islet diameter (a). Mean (± standard error, SE) number fractions are actual data from our institution (University of Minnesota) from 23 human islet preparations (high-purity, cultured fractions) prior to clinical transplantation. Mean (± SE) volume fractions are estimated from number fraction data by calculating the mean islet volumes under the assumption that the islets are spherical with a representative radius for that size range. Approximately 20% of the total number of islets were >200 *μ*m in diameter, but these islets account for ~72% of the total transplanted volume of islets (b).

**Figure 8:**
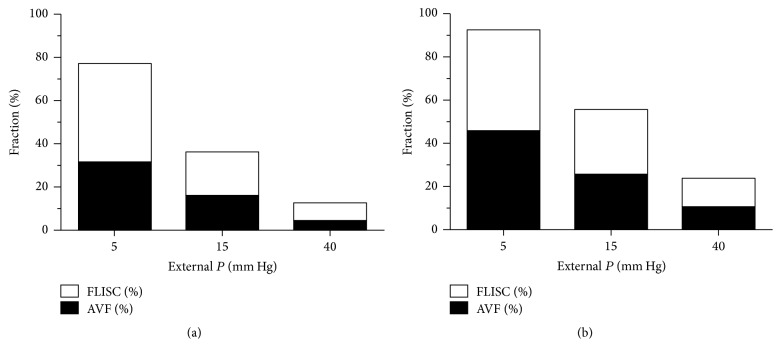
Extrapolation of model results to an entire human islet preparation, with (a) and without (b) thrombosis. Extrapolations were estimated from islet size distribution data found in Figure 7 for high-purity, cultured, alloislets. For example, to calculate the mean volume fractions for all islets of 100 *μ*m diameter, the volume fraction data for islets ranging in diameter within 50–100 and 100–150 *μ*m were averaged. AVF, anoxic volume fraction; FLISC, fractional loss of insulin secretory capacity; *P*, oxygen partial pressure.

Our model results suggest that oxygenation is important at the individual islet level but the results can also be extrapolated to an entire islet preparation. Actual size distribution data from clinical human alloislet preparations prepared at the University of Minnesota show that a small number fraction of islets (20%) are >200 *μ*m in diameter (Figure 7). When these number fractions are converted to volume fractions, these calculations indicate that despite accounting for a small fraction of the total number of islets, islets with >200 *μ*m diameter account for >70% of the total volume of an islet preparation. The AVF and FLISC calculated for an individual islet can be extrapolated to an entire islet preparation based on the actual number and estimated volume fraction data (Figure 8). Assuming that no thrombosis occurs, the AVF and FLISC for an entire islet preparation of average viability may be ~4% and >10% at the high *P*_ext_ of 40 mm Hg and >30% and >90% at the low *P*_ext_ of 5 mm Hg, respectively. When thrombus formation is assumed to occur, the AVF and FLISC for an entire islet preparation of average viability may be ~10% and >20% at the high *P*_ext_ of 40 mm Hg and >40% and >97% at the low *P*_ext_ of 5 mm Hg, respectively. These theoretical estimates indicate that a large proportion of the islet cells in a representative human alloislet preparation may be adversely affected by poor oxygenation in the early post-IT period.

## 4. Discussion

Our model results indicate the relative effect of four important parameters (islet size, islet fractional viability, local oxygen supply, and thrombosis) on the oxygenation of the early intraportally transplanted islet. Apart from *P*_ext_, islet *R* appears to have the greatest single effect on islet viability and function in any individual islet, but all of these variables are found to contribute. Thrombosis on the islet surface can also have a significant effect on oxygenation. As discussed earlier, allo-IT has not been expanded into widespread clinical use because durable insulin independence is elusive following transplant of islets from a single donor. However, it is unclear what is responsible for the gradual attrition of islets over time but it likely involves some combination of immunologic (inflammation, autoimmune recurrence or alloimmune rejection, cytokine-mediated injury, and immunosuppressant toxicity) and nonimmunologic (stress-mediated apoptosis, amyloidosis, oxygen/nutrient deprivation) causes.

Recent basic and clinical findings have suggested that gradual islet loss and dysfunction after IT exhibit features of type 2 diabetes. Like type 1 diabetes, type 2 diabetes is a disease characterized by an insufficient *β*-cell secretory capacity. However, the pathophysiologic causes of hyperglycemia are very different between the two types; type 1 diabetes results from the rapid, near-total, or total destruction of islets by the native immune system, whereas type 2 diabetes results from a gradual development of insulin resistance, a defect of insulin secretion due to chronic hyperglycemia [51–53], metabolic stress [54], and amyloid-mediated apoptosis [55]. Type 2 diabetes is believed to cause a systematic loss of islets [56] that compounds the stress on the remaining islet mass, resulting in further islet dysfunction. A recent study has shown that islets isolated from type 2 diabetic pancreata do not function normally [57]. Data indicate that an islet allograft, unless acutely rejected due to new allo- or recurrent autoimmunity, typically follows a time course representative of disease progression in type 2 diabetes. When reviewing the outcomes of major trials, the preserved C-peptide positivity that persists following IT supports the notion that most islet allografts are not overtly rejected when recipients are adequately immunosuppressed. A recent report summarized an analysis of a 52-year-old female who died of a hypertensive stroke two years after undergoing the last of two ITs on the EP and experienced gradual loss of insulin independence with sustained C-peptide secretion throughout her follow-up [58]. In the analysis, Smith et al. indicated that there was no support for an immunological explanation for the loss of both islet grafts, citing no evidence of intraislet or peri-islet inflammation on histopathologic examination of her liver and no reactivation of an islet-directed autoantibody response. Another recent report summarized histologic findings following an autopsy of a 55-year-old male who died of a myocardial infarction six months after undergoing the last of three ITs on the EP, nearly five years after the first IT, and also experienced gradual decline of islet allograft function [59], similar to the recipient in the previously mentioned report [58]. This second report noted both extracellular and intracellular amyloid deposits in >40% of the engrafted islets. Westermark et al. published a more extensive report showing similar findings from four deceased islet allograft recipients [60]. In addition to these reports, studies have shown that chronic hyperglycemia impairs islet allograft insulin secretion [61, 62] and that this defect is exacerbated when the *β*-cell mass is reduced [51]. Another recent evidence has suggested that islet allografts exhibit indicators of elevated stress-mediated apoptosis, which is enhanced with hyperglycemia [63]. These studies support a nonimmunologic basis for loss and dysfunction of the transplanted islet mass.

If late islet loss occurs in part due to nonimmunologic causes, it must be assumed that the transplanted islet mass decreases and becomes insufficient over time, which contributes to more islet loss. However, the islet doses that are currently transplanted should be adequate to reverse diabetes, at least in theory. Experience with surgical pancreatectomy has indicated that the native pancreas exhibits considerable islet reserve requiring, in some cases, the removal of >90% of the pancreas to cause overt diabetes [64]. If the native healthy adult pancreas contains 1–1.5 million islets [65, 66], roughly 100–150 thousand islets may be sufficient to prevent diabetes. It is understood that there is likely no universal minimum threshold for islet mass given the number of individual factors that impact blood glucose control (insulin sensitivity and requirements, *β*-cell regenerative capacity, etc.). However, overall experience with clinical IT has shown that the transplanted islet mass far exceeds what appears to be necessary to achieve permanent normoglycemia. Consider a 70 kg patient and assume their native pancreas contained 1 million IEs (which amounts to 14,286 IEs per kgBW). To prevent diabetes in this patient, >10% of the islet mass (~100 thousand IEs, or 1,429 IEs per kgBW) would need to remain following partial islet destruction or pancreatectomy. However, if this patient were a type 1 diabetic and a candidate for allo-IT, empirical clinical data would indicate that the minimum islet dose required to achieve insulin independence would be >60% of their original islet mass (~630,000 IEs or ~9,000 IEs per kgBW) [1–3, 6, 8] and on average would be probably higher at >70–90% (~700,000–910,000 IEs or ~10,000–13,000 IEs per kgBW) [1–3, 5, 6, 67, 68], even with the most potent induction immunosuppression currently available [69]. If this patient was a candidate for near-total or total pancreatectomy followed by auto-IT, then >35% of their original islet mass (~350,000 IEs or ~5,000 IEs per kgBW) may be required to prevent overt diabetes [70–72], and the rate of insulin independence may only be ~50–70% at 3–5 years after IT [71, 72]. In both cases, much more islets seem to be required compared to what appears to be necessary. The differences between the islet dose requirements for allo- versus auto-IT do suggest that immunologic (alloimmune rejection, autoimmune recurrence, and immunosuppressant toxicity) and nonimmunologic (brain death, prolonged cold ischemia time) factors unique to allo-IT are most likely responsible for a significant proportion of total islet loss following allo-IT. However, experiences with auto-IT indicate that perhaps >25% of the original islet mass (~250,000 IEs or ~3,500 IEs per kgBW) may be lost due to nonimmunologic factors that are present in both auto-IT and allo-IT and may contribute significantly to islet loss following intraportal IT [69–72].

There is evidence suggesting that the transplanted islet mass, which should be sufficient at the time of transplant, is reduced to levels that are inadequate due to islet loss early in the post-IT period [12, 13]. One of the possible causes for early islet loss may be inadequate oxygenation in the time preceding revascularization [10]. Of all causes of islet loss following intraportal IT, insufficient early islet oxygenation is overlooked in part due to the assumption that direct access to the blood stream should be enough to provide the necessary oxygen supply. However, there have been few attempts to confirm that this assumption is true. Studies have begun to indicate that the hepatic environment may be poor from the standpoint of oxygenation [23–26, 32, 73]. There have been two studies that have attempted to directly [32] or indirectly [26] measure the *P* near or within intrahepatically transplanted islets, and both indicate that the *P* may be very low. Carlsson et al. presented data of *P* measurements of <5 mm Hg via microelectrode probes in both transplanted islet (rat, syngeneic) and hepatic tissue and in both nondiabetic and diabetic animals [32]. It should be noted that the islets were transplanted under the liver capsule and not intraportally in their study [32]. Olsson et al. used pimonidazole, an oxygen-sensitive intracellular dye that accumulates under conditions of <10 mm Hg, to illustrate that ~70%, ~60% and ~30% of intraportally transplanted islets (mouse, syngeneic) stained positive for reduced oxygenation at 1 day, 1 month, and 3 months after IT, respectively [26]. Our model results extrapolated to entire islet preparations support the measurements by Olsson et al. The measurements by Carlsson et al. and Olsson et al. need to be considered with caution because they do not reveal much information about significant oxygen gradients that may exist in either the islet [74] or the hepatic tissue [22]. These measurements also do not provide any information regarding the longitudinal gradients that may exist along intraportal arterioles in the direction of the blood flow [75], which may vary and which would affect the oxygen availability near the intraportal islet. The model presented herein considered the data presented in Carlsson et al. and Olsson et al. and actual measurements of portal venous and hepatic arterial *P* [33], but future studies of islet oxygenation would benefit greatly from direct measurements of *P* at the level of the intraportal islet. It is important to note that measurements of available intraportal oxygen supply may be relevant not only during the early post-IT period (10–14 days), when islets rely on passive diffusion from their surfaces, but also later as there is evidence suggesting that the islets revascularize poorly (15–20% vascular density of the native islet) [23, 25] or that revascularization takes longer (>1 month) [26]. Consequently, the results of this study may highlight implications for the intraportal islet oxygenation beyond the time of revascularization and possibly indefinitely.

Islet oxygenation may have significant impact for the outcomes of auto-, allo-, and possibly xeno-IT. Our model results indicate that islet size (diameter) may have the greatest impact on the estimated AVF and FLISC. This makes sense when considering that a 300 *μ*m diameter spherical islet has 27 times the volume of a 100 *μ*m diameter spherical islet. Even though the vast majority of islets in an islet preparation are <200 *μ*m in diameter, the larger islets contribute more to the overall transplanted tissue volume. When extrapolating to an entire human islet preparation as based on actual size distribution data, and without thrombus formation, it was determined that up to 31% of the total transplanted islet volume may be anoxic along with up to 92% loss of functionality. With thrombus formation, the results are worse; up to 45% of the total transplanted islet volume may be anoxic along with up to 98% loss of functionality. The range in results is primarily attributable to the uncertainty in the actual values for available *P*_ext_. It should be noted that extrapolation of AVF and FLISC values for an entire islet preparation carries an important* caveat*: each islet experiences very different local conditions (thrombosis versus no thrombosis, low versus high *P*_ext_, etc.). These extrapolations are only meant to illustrate the potential impact of oxygenation on the scale of the entire transplanted islet volume and possibly help explain some early islet loss and dysfunction and should be studied further. There are several factors not studied in this model that would impact oxygenation, including inflammation and clustering of islets or negative oxygen flux relative to surrounding hepatic tissue. Their contributions are difficult to model and in most cases would likely worsen intraportal islet oxygenation.

In conclusion, oxygenation of the intraportally transplanted islet has not been studied extensively and may be an important contributor to islet loss and dysfunction, primarily in the early post-IT period. Future studies need to be conducted to accurately measure the intraportal *P* at the level of the engrafted islet. The liver may not be the optimal IT site and this may be in part due to poor oxygenation.

## Figures and Tables

**Table 1:** Summary of model parameters.

Description	Formulaic abbreviation	Prescribed value(s)	Units	Reference(s)
*Oxygen permeability*				
Islet	(*αD*)_1_	1.24 · 10^−14^	mol/cm/mm Hg/sec	[34, 35]
Thrombus	(*αD*)_2_	2.7 · 10^−14^	mol/cm/mm Hg/sec	[36]
*Oxygen supply*				
External blood *P*	*P*_ext_	5–40	mm Hg	[33]
*Oxygen threshold*				
Critical *P* (viability)	*P*_*C*_	0.1	mm Hg	[37, 38]
Critical *P* (function)	*P* ^*∗*^	5–15	mm Hg	[31, 34, 39–41]
*Fractional viability*				
Islet	OCR/DNA	100–300	nmol/min/mg DNA	[42–44]
*Size/thickness*				
Islet diameter	2 · *R*	100–300	*µ*m	—
Thrombus thickness	*δ*	100	*µ*m	[45–48]
*Other*				
Michaelis-Menten constant (islet OCR)	*K*_m_	0.44	mm Hg	[39]

## Abbreviations

AVF: Anoxic volume fraction
DNA: Deoxyribonucleic acid
EP: Edmonton protocol
FLISC: Fractional loss of insulin secretory capacity
IE: Islet equivalent
IT: Islet transplantation
kgBW: kg (recipient) body weight
*K*_m_: Michaelis-Menten constant for islet oxygen consumption
OCR/DNA: Oxygen consumption rate per DNA content [nmol/min/mg DNA]
*P*: Oxygen partial pressure [mm Hg]
*P*^*∗*^: Critical *P* for function (insulin secretion)
*P*_*C*_: Critical *P* for oxygen consumption and viability
*P*_ext_: External (blood) *P*
*R*: Islet radius [*μ*m]
SE: Standard error
*S*: Insulin secretion rate per unit volume of islet tissue
*S*_max_: Maximum (non-oxygen-limited) S
TNF: Tumor necrosis factor
*V*: Islet volume [*μ*m^3^]
*V*_O_2__: Oxygen consumption rate per unit volume of tissue [mol/cm^3^/sec]
(*V*_O_2__)_max_: Maximum (non-oxygen-limited) *V*_O_2__
*δ*: Thrombus thickness [*μ*m].
